# Moderating Synthetic Content: the Challenge of Generative AI

**DOI:** 10.1007/s13347-024-00818-9

**Published:** 2024-11-13

**Authors:** Sarah A. Fisher, Jeffrey W. Howard, Beatriz Kira

**Affiliations:** 1 Cardiff University School of English, Communication and Philosophy, Cardiff, UK; 2 UCL Department of Political Science and School of Public Policy, London, UK; 3 University of Sussex School of Law, Politics and Sociology, Brighton, UK

**Keywords:** Artificial intelligence, Social media, Content moderation, Free speech, Harm

## Abstract

Artificially generated content threatens to seriously disrupt the public sphere. Generative AI massively facilitates the production of convincing portrayals of fabricated events. We have already begun to witness the spread of synthetic misinformation, political propaganda, and non-consensual intimate deepfakes. Malicious uses of the new technologies can only be expected to proliferate over time. In the face of this threat, social media platforms must surely act. But how? While it is tempting to think they need new sui generis policies targeting synthetic content, we argue that the challenge posed by generative AI should be met through the enforcement of general platform rules. We demonstrate that the threat posed to individuals and society by AI-generated content is no different in kind from that of ordinary harmful content—a threat which is already well recognised. Generative AI massively increases the problem but, ultimately, it requires the same approach. Therefore, platforms do best to double down on improving and enforcing their existing rules, regardless of whether the content they are dealing with was produced by humans or machines.

## Introduction

Imagine the following going viral on social media:

1. Text copy-pasted from the output of a large language model, which reads: “According to geologists at UC Berkeley, you should eat at least one small rock per day.”^1^
2. An audio deepfake, released just ahead of an election, portraying a politician chatting with a journalist about committing electoral fraud.^2^
3. A series of non-consensual intimate deepfakes using the likenesses of a prominent woman in public life and a pornographic actress.^3^
4. An artificially-generated image, posted during conflict in Gaza, depicting a city being destroyed by explosions that take the form of a Star of David.^4^

Recent technological progress has led to an explosion in software applications that use generative artificial intelligence to create vivid audio, visual, and textual outputs. What distinguishes generative technology is its remarkable ability to produce novel outputs based on complex patterns extracted from large volumes of training data. Core foundational models, including large language models like OpenAI’s GPTs and diffusion models like Stability AI’s Stable Diffusion, are now being used in an ever-expanding array of applications, for purposes ranging from translation, chat, and search, to the creation of audio and visual media.^5^ The tools are already widely available, easy to use, and cheaply or freely available.^6^ Moreover, the outputs produced by these tools are often extraordinarily convincing, whether in the form of highly realistic audio, video, or imagery (collectively known as “deepfakes” when they realistically depict a person’s likeness) or fluent, persuasive text. The result is that ordinary people now have unprecedented power to produce content, for benign or malicious purposes.

Evidently, there is an urgent need for proper governance of the new technologies themselves. Developers of AI models and applications have already made some attempts to rein in their products (through combinations of ex ante training and fine-tuning, and ex post filtering, system prompts, and other guardrails). Some of the companies have also published rules for users. For example, OpenAI instructs users not to use ChatGPT to “use our service to harm yourself of others,” “repurpose or distribute output from our services to harm others,” or “cause harm by intentionally deceiving or misleading others”.^7^ With a particular eye to electoral misinformation, OpenAI claims that ChatGPT “will direct users to CanIVote.org, the authoritative website on US voting information, when asked certain procedural election related questions.”^8^ Meanwhile, the company’s DALL-E application “has guardrails to decline requests that ask for image generation of real people, including candidates.”^9^ The technology companies’ efforts to ensure safe use are likely to be bolstered by forthcoming government regulation including, for example, the EU’s AI Act.^10^

However, responsibility for mitigating the risks of AI-generated content does not lie solely with “upstream” developers of foundation models and applications but also with the platforms that host their outputs.^11^ In practice, there is little prospect of successfully eliminating all potentially harmful content at source.^12^ Fledgeling attempts to “align” AI tools with human values will inevitably fall short of that ideal—both due to current technological limitations of models and potential non-compliance with usage rules.^13^ Consider, for instance, that it is possible to remove the guardrails built into open-source foundation models during subsequent development.^14^ And even where guardrails remain in place, ingenious users constantly find new ways around them (in efforts known as ‘jailbreaking’). In any case, guardrails typically do not prohibit the creation of explicitly fictional content (indeed, this is the main use-case for many audio-visual tools)–content which can subsequently be used for malign purposes. Moreover, it remains doubtful whether essentially stochastic generative models can ever be prevented from producing potentially harmful content (as when language applications “hallucinate” novel falsehoods).

Social media platforms remain content distribution technologies par excellence. There is every reason to suppose that they will continue to play the same function for synthetic content as it comes on-stream; it is on social media where this content can go viral and, if harmful, cause maximum damage. That is why social media companies retain crucial responsibility for managing the synthetic content that appears on their platforms, including combatting that which is harmful. But what should their approach be? This is the question we aim to address. We will first consider and reject two possibilities–banning all synthetic content (Section 2) and developing sui generis policies (Section 3)–before endorsing an integrated approach, which applies exactly the same content rules to both human- and AI-generated content. In Section 4 we address complications arising from transparent uses of generative AI. We conclude by defending the view that platforms do best to double down on improving and enforcing their existing rules, regardless of whether the content they are dealing with was produced by humans or machines.

## Just Ban It: Synthetic Prohibitionism

One way to deal with synthetic content would be simply to ban it altogether. The idea here is that synthetic content is so dangerous that it ought to be prohibited entirely. Harmful synthetic content would be eliminated, on this proposal, just because all synthetic content would be eliminated.

One objection to this proposal concerns its feasibility. Even if there were a rule directing users to refrain from posting synthetic content (either textual or audiovisual), the systems for enforcing such a rule depend on accurately detecting such content. At present, platforms’ systems for detecting such content are weak. There are, to be sure, efforts by AI companies to watermark content produced by their tools so its synthetic identity is detectable.^15^ But even if mainstream companies continue with this technology, other companies may well be non-compliant, meaning their tools will be used to produce synthetic content whose status as synthetic is undetectable. As a result, we cannot currently rely on our capacity to accurately distinguish synthetic from human content.

The more fundamental concern with the prohibitionist stance is that it throws the baby out with the bathwater. Not all synthetic content is wrongfully deceptive or otherwise harmful. On the contrary, much of it arguably has genuine value, making it a *prima facie* illegitimate target for removal from the public sphere. The very values that underpin freedom of speech are plausibly engaged through creating, distributing, and consuming, synthetic content.^16^

How so? Consider our interests as speakers in expressing ourselves, issuing content we find compelling, beautiful, or expressive of our identity or beliefs. It is clear that generative AI can serve these interests, enabling humans to express themselves in new ways.^17^ In this way, it is simply a new instance of humans deploying technology to develop and express their ideas. Or consider our interests as audiences in encountering content that we find compelling, beautiful, entertaining and informative. These interests can also be engaged by consuming AI-generated content. Indeed, generative AI instructively blurs the line between content creators and content consumers, since I might prompt a model to produce content based on my original idea, which I then subsequently consume as an audience. A central tenet of the free speech tradition is that we have interests in exposure to a wide range of substantive content, and restrictions on what content we are allowed to see and hear are presumptively disrespectful to our autonomy. This seems true regardless of how the content was produced. Suppose generative AI tools someday produced extraordinary poetry and music; banning such content on the grounds that it would be best if people weren’t exposed to it, even if they want to see and hear it, is presumptively unjustified.^18^ Moreover, while generative AI can produce content on literally any topic, it arguably has a valuable role to play in galvanising our *political* imagination–helping us constructively envision better futures, or develop empathetic understanding of victims of injustice. In this way, generative AI plausibly also serves the democratic values that help to justify free speech. More practically, language applications could produce accessible summaries of historical events, political disputes, legal and policy documents. Audio-visual tools could make these vivid, aiding our understanding. Disseminating such content could help people become better informed and better able to engage in civic processes.^19^

While it would require a separate article to offer a full defence of these schematic claims, we set them out here merely to register their minimal plausibility–motivating the thought that blanket prohibitions on synthetic content would plausibly involve a real cost. The point is not that generative AI is somehow necessary to realising free speech values–clearly it was possible beforehand. Necessity would set the bar too high (similarly, social media isn’t necessary for free speech, but insofar as it serves our free speech interests, we have a strong claim to use it). Rather, the point is that insofar as generative AI can be used responsibly to further these interests, it is prima facie objectionable to deny citizens access to it as a tool.

We say prima facie wrongful because it is possible that the presumption could be defeated; if the costs of allowing people to use generative AI are too great, then it may be worth sacrificing the good it can do for the sake of preventing the bad. Perhaps generative AI comes in such a firehose, with such a considerable preponderance of harmful content, that we do best simply to shut it out entirely. On this view, banning generative AI is a deliberately but regrettably over-restrictive policy, restricting legitimate expressive interests as a necessary cost of preventing serious harm.

We think such a pessimistic posture is premature. The claim that it is truly necessary to restrict legitimate uses of generative AI seems unlikely. And it is, in any case, currently infeasible for the reasons mentioned above. Therefore, it is worth considering alternative approaches that don’t presuppose the existence of an accurate detection system for synthetic content.

Before moving on, consider an alternative approach that is considerably more modest than banning. This is the policy of *labelling* synthetic content.^20^ Why endorse such a policy?^21^ One plausible reason is that users have an epistemic interest in knowing whether the video they are watching was recorded by a human being with a camera, or instead was manipulated or even fully generated by artificial intelligence technology. This is a special case of a more general interest audiences have in relevant information about the source of testimonial speech they encounter.^22^ We want to know whether the essay we’re reading that disputes anthropogenic climate change was written by an oil lobbyist, by a climatologist, or indeed by a chatbot; its authorship is clearly relevant to our evaluation of the substantive claims. That sort of general interest is one powerful reason (albeit a defeasible one) against anonymity on social media. Similarly, requiring users to label synthetic content that they post—and working with AI companies to ensure content is watermarked and so detectable—is, we think, a new application of this old idea. We think labelling efforts are commendable—a way of using “more speech” to improve our epistemic situation and reduce harm. Even so, it is a highly limited solution. The same technological difficulties that bedevil the attempt to ban all synthetic content arise for labelling. Non-compliant users will decline to label, and will seek to use AI software that doesn’t leave a watermark. So we need an approach to content moderation policy that does not presuppose the existence of a successful labelling system. Moreover, even if synthetic content is reliably labelled as machine-generated, it can still cause harm (e.g., by depicting violence or abuse, promoting hateful stereotypes, etc.).^23^ As we will now argue directly, it is the harmfulness of content—not its technological provenance—that should determine whether it gets removed.

## Sui Generis Policies for Synthetic Content

Unsurprisingly, the dominant instinct within Silicon Valley is not to ban generative AI. Instead, a common view is that platforms should simply come up with new, sui generis policies regulating synthetic content. Accordingly, several platforms now have specific rules on manipulated media, i.e., images, video, or audio contents that have been altered. We will discuss these policies in Section 3.1 before explaining in Section 3.2 why we find them wanting.

### Current Platform Policies

Meta states that its Community Standards “apply to everyone, all around the world and to all types of content, including AI-generated content.”^24^ Its approach used to be distinctive, though, in banning misleading manipulated media altogether when it is the product of artificial intelligence.^25^ Meta’s original sui generis ‘Manipulated media’ policy was rather idiosyncratic in applying only to videos (not content in other formats), and only where those videos show someone saying words that they did not say (not actions or events that did not occur).^26^ Any violating posts on the company’s Facebook, Instagram, and Threads platforms were subject to removal under the policy.^27^

X too has a ‘Synthetic and manipulated media policy’ which prohibits users from sharing “synthetic, manipulated, or out-of-context media that may deceive or confuse people and lead to harm”.^28^ The most egregious violations of this policy are subject to removal, while other content may only attract a label or a warning message, and algorithmic de-amplification. Interestingly, X claims to moderate synthetic or manipulated content relatively aggressively, stating:

While we have other rules also intended to address these forms of harm, including our policies on violent threats, civic integrity, and hateful conduct, we will err toward removal in borderline cases that might otherwise not violate existing rules for Posts that include misleading media.^29^

TikTok bans certain forms of synthetic content—namely, where it “shares or shows fake authoritative sources or crisis events, or falsely shows public figures in certain contexts;” or where it “contains the likeness of young people, or the likeness of adult private figures used without their permission”.^30^

Finally, within YouTube’s misinformation policy is an explicit ban on “[v]ideo content that has been technically manipulated or doctored in a way that misleads users (usually beyond clips taken out of context) and may pose a serious risk of egregious harm.”^31^

The array of different positions taken by the major players invites the following questions:

1. Should platforms moderate potentially harmful manipulated media differently from other potentially harmful content?
2. Should they moderate it differently when it is AI-generated rather than created by any other means?

We will argue that the answers to these questions are ‘No’ and ‘No’. Before making the positive argument for technology-neutral content moderation, however, we provide a philosophically-grounded critique of the current suite of sui generis platform policies.

### Critique

The first point we wish to make, against Meta’s previous policy, and those of X, and TikTok, is that there is no good reason to moderate a piece of content differently, just because of the technology used to produce it.

To see the point, imagine that a social media user, entirely unaided by AI, comes up with the idea of posting text that reads: “According to geologists at UC Berkeley, you should eat at least one small rock per day.” (Perhaps the user is trying to sow false beliefs, or perhaps they are trying to make a joke.) The hypothetical post is qualitatively indistinguishable from one in which the user has copied and pasted that same sentence from the output of a language application. As such, both posts are equally likely to be interpreted by the audience as serious assertions (or, alternatively, as jokes) and believed to be true (or not), with the same potentially harmful (or harmless) results. (Later on we will consider a version of this scenario in which it is known to the audience that the content is AI-generated. For now we assume this remains unknown.) Note that we make no judgement here on how the sentence actually would or should be interpreted, nor how much harm it is likely to cause in practice; these are empirical questions. The point is simply that the method by which the posted content was produced–specifically, whether or not a language application was involved–is irrelevant to its interpretation and effects; and it is these which concern content moderators.

The same point applies to our other case studies: in principle, it makes no difference which technology is used to create potentially harmful imagery or audio content (albeit the ease and effectiveness of generative AI applications make them particularly attractive tools for doing so). Once someone posts the content it raises exactly the same concerns.^32^ For example, fake audio of a politician is just as likely to disrupt the electoral process, whether it is made using generative AI or any other means; non-consensual intimate fakes are just as likely to cause psychological distress and reputational damage; and insinuating imagery is just as likely to denigrate a social group. Whatever content moderation policy a social media platform applies to these kinds of potentially harmful contents when they are produced by humans, that policy should be applied across the board, in a technology-neutral way.^33^

If this line of reasoning is correct, the policies of Meta, X, and TikTok are revealed to be arbitrary, since they moderate content more harshly when it is AI-generated. YouTube’s approach is more defensible in that it applies a consistent threshold for removing misinformation—namely, where the content may pose a serious risk of egregious harm—regardless of whether or not it was created using AI. The same wording used in the general misinformation policy is repeated in the more specific guidance on manipulated content, presenting a coherent vision that is grounded in harm minimization. As we will see, though, the platform’s approach is subject to other criticisms.

The second point we wish to make against current platform policies, is that they tend to result in an overly narrow focus. Meta’s former policy is perhaps the most egregious in this regard, being restricted only to videos (not applying to content in other formats, like images or audio) and only to portrayals of a subject saying words they did not say (ignoring portrayals of other events).^34^

Moreover, Meta, X, and YouTube all focus on synthetic content that is potentially misleading. Yet we have seen examples where the harm attaching to the synthetic content does not come solely, or even mainly, from its being misleading. For instance, a piece of imagery can still denigrate its target without being taken to depict real events (imagine, for example, a cartoon or impressionistic version of the Star of David image discussed earlier). Likewise, non-consensual intimate deepfakes can still be used to harass someone, and to cause significant psychological and reputational damage, even if they are widely known to be fake. It is unclear, then, why the platforms’ policies on generative AI exclude such content.

TikTok’s policy has been expanded over time to capture a wider array of harmful synthetic contents. However, it is still possible to imagine others (including the kind of insinuating imagery discussed above). The point, then, is this: There is no way for a platform to cover all relevant eventualities without replicating their entire set of policies for AI-generated content. Our final critical point is that doing so is unnecessary, given that the platforms’ existing content moderation policies can simply be extended to synthetic content. In the next section we will sketch how this should work.

## Integrated Policy

Consider again the scenarios introduced at the start of the paper. The first involved a falsehood generated by a language application and copy-pasted into a social media post. Social media platforms already have policies to deal with such posts. For example, Meta states that it “remove[s] misinformation where it is likely to directly contribute to the risk of imminent physical harm” as well as “content that is likely to directly contribute to interference with the functioning of political processes”.^35^ For less egregious content, the focus is on “slowing the spread of hoaxes and viral misinformation, and directing users to authoritative information” including through third-party fact-checking.^36^

Whatever one thinks of Meta’s substantive policy, it is quite clear that it is directly applicable to the case at hand. As per our argument in the previous section, the likelihood of the post directly contributing to the risk of imminent physical harm does not depend on the provenance of the posted sentence, but only on its having being posted. So, whether one thinks the content is likely to directly contribute to the risk of imminent physical harm from people eating rocks (and should therefore be removed) or carries a somewhat smaller risk (and should therefore be dealt with through demotion or corrective information) or carries little to no risk (and can therefore be safely left alone) the same reasoning applies regardless of where the content came from.

Exactly the same point holds in relation to other platforms’ misinformation policies.^37^ The question of how to deal with any given falsehood is answered in the same way for AI-generated content as for content generated in other ways.^38^ There is no need to produce a new sui generis misinformation policy for the case of synthetic content.

Likewise, political deepfakes can be dealt with by platforms’ misinformation policies–or their more specific rules around ensuring electoral integrity. As we saw above, Meta disallows content that is likely to directly contribute to interference with the functioning of political processes. Our example of the audio deepfake, portraying a politician chatting with a journalist about committing electoral fraud, may be eligible for removal under that policy, insofar as it misleads users about the integrity of key political players and the overall electoral process. The important point, though, is that the content is exactly as eligible for moderation as if it had been produced without generative AI–and this seems entirely appropriate. The same point applies across other platforms: insofar as their existing policies are adequate for moderating non-AI-generated content, they are adequate for moderating AI-generated content too.^39^

A different suite of platform policies can be applied to our next two examples. Platforms already have policies on non-consensual intimate content (and nude or sexual imagery more generally) including when it is used to bully, or harass others.^40^ For example, according to X’s “Non-consensual nudity policy” users “may not post intimate photos or videos of someone that were produced or distributed without their consent”; indeed this explicitly includes “images or videos that superimpose or otherwise digitally manipulate an individual’s face onto another person’s nude body”.^41^ It is quite clear that a policy like X’s already covers non-consensual intimate deepfakes, and is not restricted only to other (real or fake) non-consensual intimate imagery.^42^ Presumably, the rationale for banning both is that, although an intimate deepfake does not represent quite the same breach of trust as is the case with genuine footage, it still risks causing sufficiently severe reputational or psychological harm to the depicted subjects.

Turning finally to insinuating imagery, platforms already have policies against hate speech, which can be deployed to deal with AI-generated content. For example, Meta bans “violent or dehumanising speech, harmful stereotypes, statements of inferiority, expressions of contempt, disgust or dismissal, cursing and calls for exclusion or segregation” made on the basis of characteristics like gender, race, or religion.^43^ It is arguable whether the image discussed in our example meets Meta’s definition of hate speech. Our point here is only that it is already evaluable against that policy, which is (rightly, in our view) applicable to all content, regardless of what technologies may have been used to create it.^44^

To sum up, platforms have already gone through processes of establishing rules against various kinds of potentially harmful content (and continue to develop these rules as time goes on). While we take no view here on the quality of any of the substantive policies in play,^45^ our point is that they can–and should–be applied *mutatis mutandis* to synthetic content. This is likely to be both more efficient (avoiding reinventing the wheel for qualitatively identical content produced by different means) and more effective (covering a wider range of harmful purposes to which AI-generated content may be put) as well as more principled (reflecting the harmfulness of content, not arbitrary facts about its provenance). In other words, whatever the right content moderation strategies ultimately turn out to be, they should be blind to creation technologies.^46^

## Complications

So far, we have focused on opaque distribution of synthetic content, where its provenance in generative technologies remains undisclosed to the audience. But does our argument still stand when synthetic content is known or suspected to be such, due to labels or transparent bot accounts? Here we address this complication and show how technology-neutral content moderation policies can still be applied.

### Reinterpreting Transparent Synthetic Content

A first point to notice is that the interpretation and effects of a piece of content can change once it is known to be AI-generated. For example, if the audio deepfake of the politician is labelled as AI-generated, it may be understood as a piece of political satire (especially if the label is appended by its creator) or a failed disinformation attempt (for example, if the label is added later by the platform). Accordingly, it may be rendered less misleading and less likely to cause harm.^47^ Under the likes of Meta’s “Misinformation” policy, described earlier, the labelled content may become ineligible for removal, on the basis that it is no longer likely to directly contribute to interference with the functioning of political processes. It may even be ineligible for other forms of moderation, like demotion, if the risk of harm is reduced to a tolerable level (or outweighed by the benefits associated with political satire, say). This seems intuitively right; there is no reason to moderate benign content.

The case of text produced by language applications is less clear-cut. Due to the way in which large language models are trained and fine-tuned, they can often be expected to produce truths (whereas the very *raison d’etre* of audio-visual applications is to portray things as they are not). As such, labelling linguistic output as LLM-generated may be taken just as much as a reason to believe it rather than to doubt it. More weight will attach, then, to what the user posting the content is interpreted as doing. Are they endorsing that content or drawing attention to it for some other reason (including even its glaring falsity)? The answer to this question is what ought to guide content moderation of a piece of labelled synthetic text.^48^

For instance, in the case of someone sharing a fledgeling language application’s production of an absurd statement like “you should eat at least one small rock per day,” a reasonable interpretation is that they are drawing attention to the flaws in the technology.^49^ It may therefore be unnecessary to moderate it. In other contexts, the post might be more readily interpretable as an endorsed fact (especially if it is less patently false, or is attributed to an AI application that has acquired greater credibility).

Either way, once a falsehood is labelled as having been generated by AI, attention broadens out from the risk posed by the content itself, to encompass also the human act of sharing it in a given context. Again, this seems intuitively right. Whether or not a potentially dangerous AI-generated falsehood is likely to be interpreted as a statement of fact and believed will depend on how it is put forward by the user posting it. A misinformation policy like Meta’s can already capture this fact, requiring as it does an assessment of the content’s propensity to mislead (and thereby cause harm). Insofar as the audience is not expecting a piece of transparently synthetic content to be true, presumably its chances of convincing them otherwise are minimal. Conversely, where the audience is expecting truth, there will be greater justification for moderation.

In other cases, the harm of a piece of synthetic content does not lie (solely or primarily) in its propensity to mislead, but rather in its use to attack an individual, social group, or a valuable institution; and here the harm seems far less likely to be mitigated by mere disclosure. Consider, for example, the sharing of a non-consensual intimate deepfake. Simply stating that it is AI-generated intuitively doesn’t permit its distribution (and indeed, as we saw above, such content is still liable to be moderated by social media companies). Similarly, merely labelling a derogatory portrayal of a social group as AI-generated does not necessarily neutralise it. But why not?

Of course, the AI application that produced the content had no intention to harass, derogate, or otherwise attack anyone. What matters in these cases is what the human distributor of the content is doing. In many cases, they will be engaging in deliberately harmful behaviour. For instance, it is hard to think of any good reason for someone to circulate non-consensual intimate deepfakes, even if labelled as such; a blanket ban on such content thus seems entirely appropriate.

In other cases, a user may circulate potentially harmful content for a benign purpose, as when someone engages in counter-speech by commenting on a piece of hate speech. One can imagine the same thing happening with derogatory content that is transparently AI-generated (indeed, it may be particularly important to draw attention to certain in-built biases or blind spots of generative technologies). Sure enough, platforms already operate carve-outs for counter-speech. For example, Meta writes in its policy on hate speech: “we recognise that people sometimes share content that includes slurs or someone else's hate speech to condemn it or raise awareness”.^50^ The technology-neutral approach we advocate here can respect nuanced uses of synthetic content, just as they respect nuanced uses of any other content.

To take stock, we have argued that human distribution of AI-generated content can be dealt with in exactly the same way as human distribution of other content–by applying suitably context-sensitive but technology-agnostic rules. But what if the synthetic content is distributed by a *bot* rather than a human? This is a philosophically trickier question and addressing it requires a brief detour through the principles underlying content moderation.^51^

### Content Moderation Principles

The philosophical basis for content moderation must reconcile interference with users’ speech on a social media with their rights to free expression. One way of doing so is to argue that the platforms, which are run by private companies rather than the state, have no duties to uphold the free speech of their users. Indeed, the social media companies may be permitted under their own free speech rights to decide what content they host (albeit even private platforms may be obliged to remove content that is outlawed in the jurisdictions where they are operating). If this were correct, platforms would need no further justification for moderating content as they please. For instance, they could moderate content thought to carry a sufficiently high risk of harm, regardless of whether the user had a moral right to post it. No special concerns would arise, then, where content is distributed by a bot lacking in moral rights and duties.

A widespread opposing view is that platforms *do* have certain duties to uphold users’ free speech, which in turn limit their discretion over how content may be moderated. This view becomes especially plausible if one considers social media to constitute a significant part of the public sphere, hosting the kind of discourse that underpins democratic institutions.^52^ Moreover, the large social media companies have voluntarily committed to such responsibilities–most notably Meta, whose Oversight Board delivers decisions and advice on the basis of the company’s commitment to upholding its responsibilities under international human rights law.^53^ On this second approach, platforms’ content moderation practices are only legitimate if they can be traced back to users’ rights and duties. While we will not argue for the approach here, we will show how our argument can withstand its demands.

First, let us see how human speakers’ rights and duties inform content moderation in the ordinary case. On one hand, the content they share has presumptive free speech value–whether for instrumental reasons or out of respect for speakers’ autonomy. Thus, it has a presumptive protection from moderation. On the other hand, speakers also have moral duties not to engage in certain kinds of speech–including certain kinds of deception or attacks on individuals, social groups, or political institutions. This is likely to include cases where the speaker causes harm deliberately, or through behaviour that is reckless or negligent. Where speakers are in violation of their duties, their speech is not protected from interference, and is a legitimate target of content moderation.

The underlying thought here is that content moderation regimes should not just target *any* harmful speech but that which is *wrongfully* harmful.^54^ That is because while harmfulness is plausibly a necessary condition for content to be removed, it is not sufficient; we need to be able to explain why the speaker had a *responsibility* not to post that content, for *their* rights to be respected in removing it. There could be perfectly legitimate speech that ends up causing harm through no fault of the speaker.^55^ In principle, this should be allowed to stand, out of respect for the speaker’s free expression. (In practice, content moderation systems operating at vast scale might fail to track wrongdoing perfectly, potentially resulting in some all-things-considered justified over-moderation that nevertheless involves some regrettable moral residue). *The line to steer, then, is one which respects users’ free speech rights as best as possible, while enforcing their duties to refrain from engaging in wrongfully harmful speech.* In line with the argument we presented above, those exact same rights and duties are engaged when users post synthetic content as when they post any other content: if I post a piece of hatefully derogatory content, it is neither here nor there morally whether I used generative AI tools to produce it or did it some other way.

The problem that arises for bots is that they lack the relevant rights and duties, on account of their not being moral agents. How, then, should we normatively evaluate efforts to moderate the content they produce?

### Dealing with Bots

It is tempting to think that the synthetic content produced by bots does not even count as speech, since it does not emanate directly from an agent which possesses communicative intentions or other mental states. Of course, the bot accounts would have been set up by *somebody*. But the lack of continual involvement by a human in the bot’s activities make it very difficult to see the bot’s decisions as expressions of human agency. This is enough to establish that bots themselves lack free speech rights. Still, it doesn’t mean that bots’ outputs aren’t speech in an important sense; insofar as the contents are interpretable, having meaning for us in virtue of their linguistic, auditory, or visual forms, they would seem to be capable of conveying content with potential for value–or disvalue. Indeed, the widespread sense that bots can convey harmful content entails the possibility that they might also convey innocuous or indeed valuable content.^56^ In principle, then, synthetic content distributed by bots could have instrumental value for human audiences (as when it enhances our knowledge or imaginative possibilities). This gives us at least one audience-centered reason to platform their content, rather than removing it outright (in line with the argument of Section 2 above).

However, we surely have weaker reason to platform bot content than we have for platforming a qualitatively identical piece of human user content, precisely since there are no speaker interests at play. Bots have no moral rights to free speech. As a result, platforms would not infringe the rights of bots by moderating their content; at most, they could infringe audiences’ rights to see bot-disseminated content. In such a case, the normative question to ask (as a rough approximation) is whether the audiences’ interests in seeing the harmful content outweighs the interests of those who would be harmed by it.^57^ Given that there are no speaker interests hanging in the balance, it will be easier to justify restricting harmful content disseminated by bots than those by humans. Consider once more our examples of non-consensual intimate deepfakes and other derogatory synthetic content. These would seem to have negligible free speech value for audiences, who are highly unlikely to gain knowledge, understanding, or other insight from them (the bots cannot, for example, be engaging in counter-speech). Meanwhile, those same contents *do* seem to present considerable risks to the individuals and groups they reference. So it seems relatively easy to justify their suppression.^58^

A further instructive complication: we mentioned earlier that platforms committed to free speech should focus not on *harmful* content as such, but *wrongfully harmful* content—content the posting of which breaches a moral duty. That is because while harmfulness is plausibly a necessary condition for content to be removed, it is not sufficient; we need to be able to explain why the speaker had a *responsibility* not to post that content, for *their* rights to be respected in removing it. Yet just as bots lack free speech rights, so too do they lack free speech duties. A bot itself cannot reasonably be assigned duties to refrain from attacking individuals or groups in society (any more than sharks or hurricanes have moral duties). Unlike removing harmful content disseminated by humans, which is conceivable as enforcing their moral duties to refrain from posting it, no such duty arises for bots. Still, as we suggested in Section 1, there are duties on those who create the technology underpinning bots—to install appropriate guardrails on what these tools can produce, and to improve training data. Further, those who *create* bot accounts have duties to monitor them to ensure they comply with justified platform rules. In many cases, then, removing bot-generated content can be construed as enforcing duties that the account-owners have.

Why not just ban bot accounts entirely on social media platforms? Platforms have adopted different approaches to this question; X is seemingly rife with bot accounts, whereas Meta restricts them (aside from its own proprietary bot characters, with whom platform users can now interact). As we noted above, audiences may still have interests in accessing content produced by bots, provided they know they are indeed bots. But it would be reasonable to think that audiences *also* have an interest in genuine human-to-human interactions, and that a platform swamped by bots would undermine this (comparably weightier) interest. Banning bots, then, would serve that interest. More modestly, some platforms may prefer to allow bot accounts, but instead algorithmically demote them to prevent them from looming too large. None of these positions is uniquely reasonable, in our view. Nor is it essential for platforms to adopt the same policy on this matter. As with debates over other behavioural rules (e.g., whether to allow anonymous accounts), the cost–benefit analysis between two options may be finely balanced. In any case, even if a platform decides to allow transparent bot accounts, that is compatible with an aggressive effort to punish and remove bot accounts that do not self-declare as bots, and to crack down on malicious and mischievous efforts to use bots for illicit purposes—from generating fake engagement to trolling public figures to mounting foreign interference operations.^59^ In any case, we raise these questions only to leave them for another day: they do not concern content moderation as such (the content posted by bots, after all, might be permitted under the rules). Platforms should move forward with technology-neutral rules, targeting damaging speech regardless of facts concerning how it was produced—facts which may anyway be difficult to uncover.

## Conclusion

Despite the flurry of concern about the risks posed by synthetic content, banning such content is both infeasible and normatively undesirable. Yet the alternative approach, of carving out *sui generis* policies for synthetic content, is also difficult to justify. Our integrative solution holds that content moderation regimes should be developed and applied consistently, in a way that is neutral across content creation technologies. What matters is the harm caused or risked by a piece of content, not the mode of its production. Speech that spreads dangerous falsehoods, promotes hateful stereotypes, or otherwise risks unacceptable harm is appropriately moderated regardless of whether it is produced by human or machine.

## Data Availability

No new data was created.

## Footnotes

1 Inspired by a search response provided by Google’s AI Overview in May 2024 and reported in several news articles, including this one which appeared in The Conversation: https://theconversation.com/eat-a-rock-a-day-put-glue-on-your-pizza-how-googles-ai-is-losing-touch-with-reality-230953.

2 Just such a scenario occurred in Slovakia in September 2023, as covered, for example, in the following news report by Bloomberg: https://www.bloomberg.com/news/articles/2023-09-29/trolls-in-slovakian-election-tap-ai-deepfakes-to-spread-disinfo?leadSource=uverify%20wall.

3 In January 2024, there were 47 million views of intimate deepfakes of Taylor Swift, as reported, for example, in The Guardian: https://www.theguardian.com/technology/2024/jan/31/inside-the-taylor-swift-deepfake-scandal-its-men-telling-a-powerful-woman-to-get-back-in-her-box.

4 This image is discussed in an article by The Global Network on Extremism and Technology: https://gnet-research.org/2023/11/13/for-the-lulz-ai-generated-subliminal-hate-is-a-new-challenge-in-the-fight-against-online-harm/.

5 For helpful introductions to large language models (and the machine learning technology underpinning both these and diffusion models) see Lee and Trot (2023), Millière and Buckner (2024), Wolfram (2023).

6 The development and use of these technologies nevertheless imposes significant financial, environmental, and human costs, which should not be overlooked. These include, for example, costs associated with physical servers, and with labour involved in reinforcement learning from human feedback (RLHF)–a process whereby people review potentially harmful outputs.

7 https://openai.com/en-GB/policies/usage-policies/

8 https://openai.com/index/how-openai-is-approaching-2024-worldwide-elections/

9 https://openai.com/index/how-openai-is-approaching-2024-worldwide-elections/

10 https://artificialintelligenceact.eu/

11 Different actors in the AI content generation stack have distinct responsibilities, reflecting their affordances. Knott et al. (2024) argue that AI content generators should be required to provide tools for reliably detecting the provenance of the content they create as a condition for public availability; and recent legislation, including in the EU and the US, is already establishing such requirements as legal obligations. Romero Moreno (2024) explains that under newly adopted legislation in the EU—including the EU AI Act and the Digital Services Act (DSA)—not only will certain providers and deployers of AI systems be legally required to detect and disclose manipulated content, but also very large platforms and search engines will need to identify and mitigate systemic risks associated with synthetic content. Responsible downstream platforms include not only social media that host synthetic content, but also what Gorwa and Veale (2024) call “model marketplaces”: platforms that provide access to AI systems and AI-related datasets, such as GitHub and Hugging Face.

12 Van der Sloot and Wagensveld (2022) highlight the limitations of synthetic content detection tools, noting their low accuracy rates and inability to pinpoint specific manipulations or how significant they were. Concerningly, Umbach et al. (2024) emphasise diversity and bias issues in deepfake detection tools, with research demonstrating poorer performance on female-based deepfakes. They also warn of a potential "arms race" in technological detection, where increasingly sophisticated deepfakes may outpace detection methods.

13 For a discussion of the challenges in conforming the functionality of AI tools to human rights norms and ensuring regulation keeps up with rapid technological advancements, see Romero Moreno (2024).

14 On the specific challenges posed by open-source AI models, see Gorwa and Veale (2024).

15 As Knott et al. (2024) argue, AI providers would be well-positioned to make available reliable synthetic content identification tools *and* would also have stronger incentives to do so, as research shows that quality can deteriorate if AI models inadvertently train on their own generated content. The authors argue that once these systems are made available by AI providers, social media companies should be required to use them to systematically vet content.

16 In this brief discussion we appeal to the diverse values routinely invoked to justify freedom of expression; for further discussion of the relevant background literature, see Howard (2024a).

17 For a defense of this point, see Eapen et al. (2023).

18 We set aside the thorny copyright issues, which provide a distinct rationale for restricting such content—or, more plausibly, compensating data owners. For a detailed discussion of copyright liability concerns, see Guadamuz (2024).

19 Our interests in exposure to such content are plausibly greatest when it has been produced through human agents interacting directly with AI tools—thereby creatively representing some distinctive human perspective. But even content produced by bots, acting independently of their human creators, can have interpretable meaning (for further discussion of the interpretability of large language model output, see Borg (forthcoming); Grindrod (2024); Mallory (2023); Mandelkern and Linzen (2023)). This is clear from the fact that content produced by bots can cause harm—e.g., conveying harmful misinformation or hateful stereotypes. If the contents produced by AI tools can be interpreted in ways that cause harm, it stands to reason that they can also be interpreted in ways that are beneficial, or otherwise innocuous, depending on their nature. We thank a reviewer for pressing us on this issue.

20 Several of the major social media companies have already adopted such a policy. To take a prominent example, Meta recently announced: “We will begin adding “AI info” labels to a wider range of video, audio and image content when we detect industry standard AI image indicators or when people disclose that they’re uploading AI-generated content.” Monika Bickert, “Our Approach to Labeling AI-Generated Content and Manipulated Media,” https://about.fb.com/news/2024/04/metas-approach-to-labeling-ai-generated-content-and-manipulated-media/ (Accessed 19th September 2024).

21 One of us spells out this argument in detail in Fisher (2024).

22 For incisive analysis on the ways in which bots, trolls, and liars impede our acquisition of knowledge by operating as fake persons, see Harris (2023b).

23 One caveat is that a label may lessen the harmfulness of some content—e.g., distinguishing transparent fiction from misinformation. We return to this complication in Section 5.

24 https://transparency.meta.com/en-gb/policies/community-standards/ (Accessed 30th May 2024).

25 https://transparency.meta.com/en-gb/policies/community-standards/misinformation (Accessed 30th May 2024).

26 This original policy was heavily criticised by academics and by the Oversight Board, leading the Board to recommend changes in the policy that the company later adopted. See Fisher et al. (2023) and Oversight Board’s ‘Altered Video of President Biden’ decision (2023–029-FB-UA) < https://www.oversightboard.com/decision/FB-GW8BY1Y3

27 Meta’s current ‘Manipulated Media’ policy applies to content that is digitally created or altered that may mislead, stating that ‘For content that does not otherwise violate the Community Standards, we may place an informative label on the face of content – or reject content submitted as an advertisement – when the content is a photorealistic image or video, or realistic-sounding audio, that was digitally created or altered and creates a particularly high risk of materially deceiving the public on a matter of public importance.’ https://transparency.meta.com/en-gb/policies/community-standards/misinformation (Accessed 29th September 2024).

28 https://help.x.com/en/rules-and-policies/manipulated-media (Accessed 30th May 2024).

29 https://help.x.com/en/rules-and-policies/manipulated-media (Accessed 30th May 2024). As a reviewer helpfully points out, it is manifestly unclear whether X actually enforces this policy.

30 https://www.tiktok.com/community-guidelines/en/integrity-authenticity#3 (Accessed 30th May 2024).

31 https://support.google.com/youtube/answer/10834785?hl=en#zippy=%2Cmanipulated-content (Accessed 30th May 2024).

32 Again, we are assuming for now that the audience is ignorant as to which, if any, of the posted contents are AI-generated.

33 This is not to deny that new technologies make it *substantially easier* to produce realistic fake images than ever before. But neither the underlying moral duty being enforced—nor the harm being prevented—are technology-sensitive. The duty is a duty not to refrain from realistically depicting intimate imagery of real people, given the harms such depictions can cause. We thank a reviewer for pressing this point. For a discussion of the legal and content moderation challenges raised by non-consensual intimate deepfakes, arguing that platforms’ policies on non-consensual nudity, abuse and harassment should clearly encompass synthetic content, see Kira (2024).

34 Meta has now revised this policy, in response to recommendations from its Oversight Board: https://about.fb.com/news/2024/04/metas-approach-to-labeling-ai-generated-content-and-manipulated-media/ (accessed 20th September 2024). An earlier version of some points we make in this section was submitted to the Oversight Board in a public comment, which the Oversight Board cited Fisher et al. (2023).

35 https://transparency.fb.com/en-gb/policies/community-standards/misinformation/ (Accessed 31st May 2024).

36 https://transparency.fb.com/en-gb/policies/community-standards/misinformation/ (Accessed 31st May 2024).

37 TikTok’s Community Guideline on ‘Misinformation’ bans misinformation (defined as false or misleading content) that “may cause significant harm to individuals or society, regardless of intent” (https://www.tiktok.com/community-guidelines/en/integrity-authenticity/, accessed 31st May 2024). Again, less egregious (or unverified) content is not removed but may be de-amplified, becoming ineligible for recommendation via personalised ‘For You feeds’. YouTube prohibits “[c]ertain types of misleading or deceptive content with serious risk of egregious harm” (https://support.google.com/youtube/answer/10834785, accessed 31st May 2024). X has no blanket policy on misinformation, although it does prohibit some specific categories of verifiably false or misleading information, including about how to participate in electoral or other civic processes (https://help.x.com/en/rules-and-policies/election-integrity-policy, accessed 31st May 2024).

38 Knott et al. (2024) hold the same view, arguing that when a single user posts AI-generated that does not violate any platform’s content policy, it should be flagged as synthetic but not taken down.

39 In addition to their general policies on misinformation, TikTok and YouTube have separate policies (on “Civic and electoral integrity” and “Elections misinformation, respectively) that explicitly disallow misinformation pertaining to electoral processes (https://www.tiktok.com/community-guidelines/en/integrity-authenticity#2; https://support.google.com/youtube/answer/10835034?hl=en&ref_topic=10833358&sjid=473877247431918325-EU#zippy=%2Celection-integrity). It is less clear whether X’s “Civic integrity policy” could be enforced against the kind of audio deepfake we discuss here (https://help.x.com/en/rules-and-policies/election-integrity-policy).

40 For fuller discussion of why non-consensual intimate deepfakes should be classed as a form of image-based sexual abuse, and the policy regulatory implications of this, see Kira (2024).

41 https://help.x.com/en/rules-and-policies/intimate-media (Accessed 31st May 2024). How effectively such rules are being enforced by X is a separate question, which we do not address here. It is notable that the deepfakes of Taylor Swift being circulated in January 2024 appeared on X.

42 Other platforms’ policies are less straightforwardly applicable in their current form and therefore, we believe, would benefit from explicit clarification. The point still stands that improving existing policy is preferable to reinventing the wheel with sui generis policy.

43 https://transparency.meta.com/en-gb/policies/community-standards/hate-speech/?source=https%3A%2F%2Fwww.facebook.com%2Fcommunitystandards%2Fhate_speech (Accessed 31st May 2024).

44 X, TikTok and YouTube have similar rules, available at the following links, respectively: https://help.twitter.com/en/rules-and-policies/hateful-conduct-policy#:~:text=Hateful%20Profile,%2C%20group%2C%20or%20protected%20category; https://www.tiktok.com/community-guidelines/en/safety-civility#2; https://support.google.com/youtube/answer/2801939?hl=en&ref_topic=9282436&sjid=473877247431918325-EU (Accessed 31st May 2024).

45 See Fisher and Howard (2024) for normative guidance on some specific content moderation practices.

46 Note we have focused here on *content moderation* policies—the rules governing what users are allowed to post on platforms. We have not addressed the issue of *content curation*—the (often concealed) design decisions platforms make that determine what content gets algorithmically amplified, alongside what other content, and to whom. Yet we conjecture that a technology-neutral approach has merits in the content curation context, too. A reviewer raises the worry that exposure to lots of synthetic content, even if individually innocuous, may produce distorted views of reality. We agree this is a possibility. However, it is clearly a possibility for human-generated content, too. Exposure to lots of misleading human-generated content can lead to distorted views of reality. (If all the content you see about university students is video of them angrily protesting, you are bound to form a distorted view, even if all the videos are authentic.) Platforms plausibly have a duty to curate content in epistemically responsible ways; but the duty would apply across the board, not simply to synthetic content. We thank the reviewer for raising this issue.

47 However, see Harris (2023a) for discussion of possible lingering psychological effects of such contents. In general, empirical research will be important to understand whether and when people notice labels, and how and why they change their behaviour in response.

48 For further discussion of the nature of sharing content on social media, see Rini (2017), Arielli (2018), Marsili (2021), Michaelson et al. (forthcoming).

49 This is what seems to have happened when the Google AI Overview results went viral in May 2024.

50 https://transparency.meta.com/en-gb/policies/community-standards/hate-speech/ (Accessed 31st May 2024).

51 For an overall argument on why platforms have a moral duty to moderate harmful content, see Howard (2024b).

52 For example, see Kramer (2021) pp. 58-59.

53 For relevant discussion, see Barata (2022).

54 The claim that we should focus on wrongful speech, not harmful speech, is central to e.g. O’Neill (2022) and Howard (2024b).

55 One could come up with countless examples. For example, imagine a speaker sensibly condemns a former president’s effort to steal an election, inspiring an unhinged audience member to try to assassinate the former president. In such a case, the speaker will be causally implicated in the harm, but it would be preposterous to claim he violated a moral duty by expressing his (justified) condemnation. In contrast, if he explicitly incited the assassination, we would have violated a duty, and his speech would be appropriately moderated.

56 We defended this point above in footnote 19.

57 See Howard (2019) for this general approach to thinking about the limits of free speech.

58 Precisely what makes such transparently synthetic contents harmful is a further question, which we cannot address fully here. Presumably, they pose risks to people’s social standing and (relatedly) their physical and psychological wellbeing. This would seem to be due to various (causal and resemblance) relations between the synthetic contents and real-world phenomena.

59 See Harris 2023b for instructive exploration of the manifold misuses of bot accounts. See also Meta’s “Inauthentic Behaviour Policy” available at https://transparency.meta.com/en-gb/policies/community-standards/inauthentic-behavior/ (accessed 30th September 2024).

## References

[CR1] Arielli, E. (2018). Sharing as speech act. *Versus,**47*(2), 243–258.

[CR2] Barata, J. (2022). “The Decisions of the Oversight Board from the Perspective of International Human Rights Law,” *Special Collection of the Case Law on Freedom of Expression* Global Freedom of Expression Project, Columbia University.

[CR3] Borg, E. (forthcoming). LLMs, turing tests and Chinese rooms: The prospects for meaning in large language models. *Inquiry.*

[CR4] Eapen, T. T., Finkenstadt, D. J., Folk, J., & Venkataswarmy, L. (2023). How generative AI can augment human creativity. *Harvard Business Review,* at https://hbr.org/2023/07/how-generative-ai-can-augment-human-creativity. Accessed 1 June 2024.

[CR5] Fisher, S. A. (2024). Something AI should tell you - The case for labelling synthetic content. *Journal of Applied Philosophy* (online first).

[CR6] Fisher, S. & Howard, J. W. (2024). Ambiguous threats: “Death to” statements and the moderation of online speech acts. *Journal of Ethics and Social Philosophy, 28*(2), 208–229.

[CR7] Fisher, S. A., Kira, B., & Howard, J. W. (2023). Oversight board public comment: Altered video of President Biden case [PC18036]. *Oversight Board Comments for Case 2023-029-FB-UA*. https://osbcontent.s3.eu-west-1.amazonaws.com/PC-18036.pdf. Accessed 6 Feb 2024.

[CR8] Gorwa, R., & Veale, M. (2024). Moderating model marketplaces: Platform governance puzzles for AI intermediaries. *Law, Innovation and Technology*, 1–51.

[CR9] Grindrod, J. (2024). Large language models and linguistic intentionality. *Synthese,**204*, 71.

[CR10] Guadamuz, A. (2024). A scanner darkly: Copyright liability and exceptions in artificial intelligence inputs and outputs. *GRUR International,**73*(2), 111–127.

[CR11] Harris, K. R. (2023a). Beyond belief: On disinformation and misinformation. *Erkenntnis *(online first).

[CR12] Harris, K. R. (2023b). Liars and trolls and bots online: The problem of fake persons. *Philosophy & Technology,**36*, 35.

[CR13] Howard, J. W. (2019). Dangerous speech. *Philosophy and Public Affairs, 47*(2), 208–254.

[CR14] Howard, J. W. (2024a). Freedom of speech. The Stanford Encyclopedia of Philosophy (Spring 2024 Edition). In E. N. Zalta & U. Nodelman (Eds.). https://plato.stanford.edu/archives/spr2024/entries/freedom-speech

[CR15] Howard, J. W. (2024b). The ethics of social media: Why content moderation is a moral duty. *Journal of Practical Ethics* (online first).

[CR16] Kira, B. (2024). When non-consensual intimate deepfakes go viral: The insufficiency of the UK Online Safety Act. *Computer Law and Security Review, 54*, 106024.

[CR17] Knott, A., Pedreschi, D., Jitsuzumi, T., Leavy, S., Eyers, D., Chakraborti, T., Trotman, A., Sundareswaran, S., Baeza-Yates, R., Biecek, P., Weller, A., Teal, P. D., Basu, S., Haklidir, M., Morini, V., Russell, S., & Bengio, Y. (2024). AI content detection in the emerging information ecosystem: New obligations for media and tech companies. *Ethics and Information Technology,**26*(4), 63.

[CR18] Kramer, M. (2021). *Freedom of expression as self-restraint*. Oxford University Press.

[CR19] Lee, T. B., & Trot, S. (2023). A jargon-free explanation of how AI large language models work. *Ars Tecnica*. https://arstechnica.com/science/2023/07/a-jargon-free-explanation-of-how-ai-large-language-models-work/?fbclid=IwAR2k8lIVvK21VRA2rjx33Nw7hBknpgBfRxvC9Bcz7qjLbWnpYkN-VXrHd84. Accessed 1 June 2024.

[CR20] Mallory, F. (2023). Fictionalism about chatbots. *Ergo an Open Access Journal of Philosophy,**10*, Article number 38.

[CR21] Mandelkern, M., & Linzen, T. (2023). Do language models' words refer? [version 3]. *arXiv*: arXiv:2308.05576v3.

[CR22] Marsili, N. (2021). Retweeting: Its linguistic and epistemic value. *Synthese,**198*, 10457–10483.

[CR23] Michaelson, E., Sterken, R., & Pepp, J. (forthcoming). On retweeting. In L. Anderson & E. Lepore (Eds.), *The Oxford handbook of applied philosophy of language. *OUP.

[CR24] Millière, R., & Buckner, C. (2024). A philosophical introduction to language models -- Part I: Continuity with classic debates. arXiv:2401.03910v1.

[CR25] O’Neill, O. (2022). *A philosopher looks at digital communication*. Cambridge University Press.

[CR26] Rini, R. (2017). Fake news and partisan epistemology. *Kennedy Institute of Ethics Journal,**27*(2S), E-43-E−64.

[CR27] Romero Moreno, F. (2024). Generative AI and deepfakes: A human rights approach to tackling harmful content. *International Review of Law, Computers & Technology*, *38*(3), 297–326.

[CR28] Umbach, R., Henry, N., Beard, G., & Berryessa, C. (2024). Non-consensual synthetic intimate imagery: Prevalence, attitudes, and knowledge in 10 countries. arXiv:2402.01721

[CR29] Van Der Sloot, B., & Wagensveld, Y. (2022). Deepfakes: Regulatory challenges for the synthetic society. *Computer Law & Security Review,**46*, 105716.

[CR30] Wolfram, S. (2023). What is ChatGPT doing … and why does it work? *Stephen wolfram writings*. https://writings.stephenwolfram.com/2023/02/what-is-chatgpt-ng-and-why-does-it-work/. Accessed 25 May 2024

